# Gruel as an Infants' Food

**Published:** 1899-08-19

**Authors:** 


### GRUEL AS AN INFANTS' FOOD.

Notwithstanding all that has been said against the use of starchy materials in the feeding of babies there still are many people, especially mothers and old style nurses, who, approaching the subject from the practical rather than the theoretical side, are ready to declare that a little "thickening" in the milk makes it "suit" children better than when it is administered plain or merely diluted with water. We have long been accustomed to regard this as due to its effect in altering the character of the curd, so that instead of being massive and tenacious, as the curd of cows' milk naturally is, it becomes flocculent, non-adhesive, and easily digested, and it has long been the custom to order that, in preparing food for infants who fail to digest cows' milk, the milk should be diluted with barley-water. The barley-water has been looked upon as a comparatively innocuous substance, by the addition of which certain evils connected with the digestion of cows' milk can be overcome, and therefore it has been ordered, but it has still been generally maintained that this material, being of a starchy nature, and being therefore incapable of digestion by the young infant, is not to be looked upon as a food.

Dr. Henry Dwight Chapin, of New York, has recently, however, gone over the whole ground afresh, and his results—as given in a paper read before the American Pediatric Society[1]—are of very considerable interest, for they tend to support the "old woman's tales" as to the utility of gruels, and to throw doubt upon much that has been laid down with great positiveness in regard to the inappropriateness of carbohydrate food for the young infant.

After detailing the experiments by which he had shown the correctness of the old idea that the addition of a decoction of cereals, in this case barley water, alters the character of the curd and renders it easier of digestion, he goes on to say: "Besides attenuating the casein, the addition of gruel to cows' milk increases the nutritive value of the food. This is of great service in that class of cases in which bottle-fed babies show stationary or losing weight. . . . . A proper addition of gruels to milk will not infrequently check wasting." The final decision in any therapeutic question must, of course, rest upon clinical experience, and Dr. Chapin says that he has tried all kinds of infant feeding with that hardest class of cases, bottle-fed babies, in hospital and dispensary practice, and that the best results are obtained by adding gruels to the milk. The theory is that the cereal helps to attenuate the curd of the cows' milk, and what happens in practice is that the infant is not so apt to vomit thick curds, and the tendency to loss of weight is lessened. But he adds that Jacobi, with his long clinical experience, not only maintains the value of decoctions of the cereals as an addition to cows' milk, but finds that even very young infants thrive better when cows' milk is diluted with gruels than when a mere sugar solution is added. In Germany, Heubner, of Berlin, comes to the same conclusion from a wide clinical experience. The common objection advanced against this mode of feeding is, he says, that the digestive power of the nursling cannot cope with starchy food in any form, but "while large quantities of starch should be withheld in infancy, even the youngest baby can tolerate and digest a small and proper amount," for, according to Hammersten,[2] ptyalin, or salivary diastase, the amylolytic ferment of the saliva, occurs in new-born infants. But he shows that any disadvantage in the employment of wheat or barley flour, from the starch contained in these cereals, can be easily overcome by dextrinising the gruels. An easy, rapid, and simple method of doing this is by the addition of diastase. Most of the commercial malt extracts are sufficiently active in diastase to produce the desired effect, but he prefers the use of diastase itself as being both speedy, efficient, and also cheap. It may be made as follows:

"A tablespoonful of malted barley grains, crushed, is put in a cup and enough cold water added to cover it, usually two tablespoonfuls, as the malt quickly absorbs some of the water. This is prepared in the evening and placed in the refrigerator over night. In the morning the water, looking like thin tea, is removed by a spoon or strained off, and is ready for use. About a tablespoonful of this solution can be thus procured and is very active in diastase. It is sufficient to dextrinise a pint of gruel in ten or fifteen minutes." The gruel may be prepared in the following manner: "A tablespoonful of wheat flour or barley flour is beaten up into a thin paste with a little cold water and then stirred in a pint of water, which is boiled for fifteen minutes. When cool enough to be tasted, a tablespoonful of the above solution or a teaspoonful of malt extract or preparation of diastase is added, and the mixture stirred as further cooling takes place. The great bulk, if not all of the starch, will be thus dextrinised in about fifteen minutes." A gruel prepared as above may be used as a diluent of cows' milk, and can, he says, be assimilated by the youngest and weakest infant. If the bowels tend to looseness wheat or barley flour may be used, but when there is constipation oatmeal had better be employed. Barley flour is difficult to obtain except in the form of the prepared barley sold by some makers. But the barley grains may be used. A tablespoonful may be soaked overnight in a little cold water, and then, this being removed, the barley may be boiled for five or six hours in a pint of water, replenishing the water as it evaporates. It is worth remembering that in hot weather, when it is sometimes necessary to abandon milk of all kinds for a time, simple dextrinised gruels form a nourishing drink for babies.

[1] New York Med. Rec., Aug. 5. [2] Physiological Chemistry.

